# Prevalence of *Cryptosporidium parvum*, *Giardia duodenalis* and *Eimeria* spp. in diarrhoeic suckling calves from north-western Spain and analysis of their interactions

**DOI:** 10.1080/23144599.2024.2447172

**Published:** 2025-01-09

**Authors:** Cynthia López-Novo, Seila Couso-Pérez, Alberto Prieto, Jose Manuel Díaz-Cao, David García-Dios, Gonzalo López-Lorenzo, Susana Remesar, Elvira Ares-Mazás, Ceferino López, Patrocinio Morrondo, Hipólito Gómez-Couso, Pablo Díaz

**Affiliations:** a Galicia (Grupo INVESAGA). Departamento de Patología Animal. Facultad de Veterinaria, Universidade de Santiago de Compostela, Lugo, Spain, Investigación en Sanidad Animal; b Grupo Interdisciplinar en Tecnología Farmacéutica, Inmunobiología Parasitaria y Parasitosis Hídricas (PARAQUASIL). Departamento de Microbiología y Parasitología. Facultad de Farmacia, Universidade de Santiago de Compostela, Santiago de Compostela, A Coruña, Spain; c Instituto de Investigación del Medio Acuático para Una Salud Global (IARCUS), Universidade de Santiago de Compostela, Santiago de Compostela, A Coruña, Spain; d IBADER- Instituto de Biodiversidade Agraria e Desenvolvemento Rural, Universidade de Santiago de Compostela, Lugo, Spain

**Keywords:** Enteric protozoa, co-infection, cattle, neonatal diarrhoea, Galicia

## Abstract

Although *Cryptosporidium parvum*, *Giardia duodenalis* and some *Eimeria* species are frequently involved in neonatal calf diarrhoea (NCD), detailed studies on their interactions are scarce. Therefore, a cross-sectional study including faecal samples from 404 diarrhoeic calves aged 0–30 days was performed. *Cryptosporidium* oocysts and *G. duodenalis* cysts were detected by immunofluorescence antibody test and positive samples were molecularly characterized. *Eimeria* oocysts were microscopically detected using the modified McMaster technique and morphometric species identification was performed. *Cryptosporidium* infections (53.7%) predominated over those by *G. duodenalis* (12.4%) and *Eimeria* spp. (6.9%). Three *Cryptosporidium* species were identified: *C. parvum* (98.0%), *C. bovis* (1.0%) and *C. ryanae* (1.0%). Eleven *C. parvum* zoonotic subtypes were detected, with IIaA15G2R1 as the most common; only *G*. *duodenalis* assemblage E was identified. Moreover, 10 *Eimeria* species were found, being *Eimeria ellipsoidalis* (96.4%), *Eimeria bovis* (85.7%), and *Eimeria zuernii* (71.4%) predominant. A significant positive association between *G. duodenalis* and *Eimeria* spp. was detected as well as a negative association between *C. parvum* and *Eimeria* spp. Our results showed that *C. parvum* wasprevalent in diarrhoeic calves under 21 days of life; *Eimeria* spp. and *G. duodenalis* were especially common in animals in their fourth week of life. Concurrent infections increased with age. Moreover, the study also revealed potential public health risks since a noticeable percentage of animals were infected with *C. parvum* zoonotic subtypes. Further studies are needed for determining the role of these parasites in co-infections with other enteropathogens and their implications in the pathogenicity of NCD.

## 1. Introduction

Neonatal calf diarrhoea (NCD) is the main cause of morbidity in calves in their first month of life, resulting in weight loss, delayed growth, and high rates of mortality. Thus, it is considered one the major causes of economic loss for cattle farms worldwide [1]. The emergence of a NCD outbreak in a herd is the consequence of the interaction of several factors including the animal immune status, management conditions and multiple bacterial, viral and parasitic enteropathogens, which are most frequently transmitted by the faecal-oral route [1]. The protozoan parasites of the genera *Cryptosporidium*, *Giardia* and *Eimeria* are considered the major parasites involved in the NCD syndrome worldwide [2,3].

Around 20 species and genotypes of *Cryptosporidium* have been identified in cattle, being *Cryptosporidium andersoni*, *Cryptosporidium bovis, Cryptosporidium parvum* and *Cryptosporidium ryanae* the most common species [4,5]. The zoonotic *C. parvum* is the dominant species in neonatal calves in most countries [6–9]. This species is highly pathogenic, being considered a common and serious primary cause of NCD outbreaks in cattle farms [9]. Moreover, some *C. parvum* subtypes, especially those belonging to the allelic families IIa and IId, have also been recognized as important human pathogens and were involved in most cryptosporidiosis outbreaks reported in humans [5].

The flagellate *Giardia duodenalis* (syn. *Giardia intestinalis*, *Giardia lamblia*) is a common protozoan in cattle, especially in animals under six months [10–12]; nevertheless, its role in the NCD syndrome is controversial [13–15]. *Giardia duodenalis* is considered a species complex comprising eight assemblages with different host specificity [12]. Although assemblage E predominates in cattle, the less host-specific assemblages A and B were also identified in this host and assemblages C, D and F, which are mainly detected in canids and felids, respectively, have also been sporadically reported in cattle [10,12]. Humans are usually infected with assemblages A and B, though cases of infection with assemblage E have been occasionally reported and the zoonotic potential of this genotype needs further assessment [10].

A total of 13 highly host-specific and non-zoonotic *Eimeria* species are considered valid in cattle, but only a few are pathogenic [16]. Thus, *Eimeria bovis* and *Eimeria zuernii* are related to severe haemorrhagic diarrhoea in calves; nevertheless, *Eimeria alabamensis*, *Eimeria auburnensis* and *Eimeria ellipsoidalis* are considered as moderate-low pathogenic species [3,16].

Due to the significant impact of cryptosporidiosis and giardiosis on animal and public health, several epidemiological investigations have been performed in both diarrhoeic and asymptomatic cattle from Spain in the last 30 years [4,7,8,11,17–22]. In contrast, information on *Eimeria* infections is restricted to two studies [22,23]. In diarrhoeic neonatal calves from Spain, available data indicates that *Cryptosporidium* is very prevalent (49.2–64.7%) [7,8,11,17–19,21,22]; on the contrary, investigations on the presence of *G. duodenalis* and *Eimeria* spp. in NCD showed that both protozoans are involved in less than 10% of the cases [11,22]. In addition, studies on the interactions between these three enteropathogens in diarrhoeic neonatal calves are very scarce, even though this data is essential for understanding their role in this syndrome. In fact, most of these studies do not include genotypic/subtype analyses or even specific identification of the detected pathogens [11,14,22,24,25], hampering the determination of their pathogenic and zoonotic potential. The aim of the present study was to update data on the prevalence of *Cryptosporidium* spp., *G. duodenalis* and *Eimeria* spp. in diarrhoeic calves younger than one month from north-western Spain, and analyse their interactions as well as the possible influence of age, faecal consistency and seasonality on the prevalence of infection and (oo)cyst shedding. In addition, the zoonotic potential of samples positive for *C. parvum* and *G. duodenalis* was molecularly determined.

## 2. Materials and methods

### 2.1. Study area and sample collection

This study was carried out in Galicia (north-western Spain), one of the most important cattle rearing areas in this country. In this region, most cattle herds are small (79.1% of farms have less than 30 animals) with semi-extensive rearing systems [26].

In order to estimate the apparent prevalence of each protozoan with a 95% confidence interval and 95% precision, the sample size was calculated using Epitools (https://epitools.ausvet.com.au). The minimum sample size established was 385, considering the highest sample size obtained from data previously reported in diarrhoeic calves in Spain [7,8,22]. During a four-year period (2017–2020), a total of 404 faecal samples from diarrhoeic calves younger than one month old were collected by veterinary practitioners in 221 cattle farms. In addition, information on the farm of origin and the age of the animal was gathered. Two seasons were considered according to Boullosa et al. [27]: warm season (weeks 20–40 of the year) and cold season (rest of the year). Faecal samples were kept at 4°C and processed within 48 h after collection. In the laboratory, faecal consistency was scored as semi-liquid or watery [28].

### 2.2. Microscopic detection of protozoans

A diphasic concentration method using distilled water and ethyl acetate (4:1) was applied on 2 g of each faecal sample in order to concentrate *Cryptosporidium* and *G. duodenalis* (oo)cysts as previously described [29]. Then, 10 μl of each concentrated sample was analysed using an immunofluorescence antibody test (IFAT) (Aqua Glo G/C, Waterborne Inc., New Orleans, LA, USA), following the manufacturer’s instructions. The presence of *Cryptosporidium* and *G. duodenalis* (oo)cysts was detected and quantified in 20 non-overlapping randomly chosen microscopic fields at 200 × magnification using a fluorescence microscope (CX43, Olympus, Tokyo, Japan). The (oo)cyst shedding score was calculated according to the average number of parasitic forms per field at 200 × magnification as follow: 1 [0–1 (oo)cysts], 2 [>1–5 (oo)cysts] and 3 [>5 (oo)cysts] [30].

*Eimeria* spp. oocysts were detected and quantified by the modified McMaster technique using saturated saline solution (specific gravity 1.19 g/mL) [31]; the detection limit was 50 oocysts per gram (opg) of faeces. *Eimeria*-positive samples were selected for further species differentiation. Thus, oocysts were firstly sporulated by incubation in a 2.5% (w/v) potassium dichromate solution (K_2_Cr_2_O_7_, Sigma-Aldrich, St. Louis, MO, USA) for 7 days at room temperature. Finally, a maximum of 100 oocysts per sample were measured under a bright field microscopy (microscope CH-2, Olympus) to perform species identification using previously reported morphometric keys [32]. This information was used for calculating the opg values for *E. bovis* and *E. zuernii*.

### 2.3. *Molecular characterization of* Cryptosporidium *spp. and* Giardia duodenalis

Those samples which were IFAT-positive for *Cryptosporidium* spp. and/or *G. duodenalis* were selected for further molecular analysis. DNA was extracted from 200 mg of the sediment of concentrated samples using the QIAamp Fast DNA Stool Mini Kit (Qiagen, Valencia, CA, USA) according to the manufacturer’s instructions. All samples were subjected to an initial step involving three freeze-thaw cycles [7].

For identifying the *Cryptosporidium* species, a nested PCR targeting the small ribosomal subunit (*SSU rRNA*) gene and restriction fragment length polymorphism (RFLP) analysis with the endonucleases *SspI*, *VspI,* and *MboII* (New England Biolabs, Ipswich, MA, USA) were performed using previously described primers and protocols [33,34]. PCR products and restriction fragments were subjected to electrophoresis in 1% or 2% agarose gels, respectively, and visualized in a GelDoc Go imaging system (Bio-Rad, Hercules, CA, USA) after staining with RedSafe (INTRON Biotecnology, Gyeonggi, South Korea). The species/genotype assignment was performed by comparing the RFLP profiles with known ones reported in literature [33]. A subset of representative positive isolates, including at least one of each *Cryptosporidium* species/genotypes identified, was sequenced to confirm the RFLP results. Sequencing was performed at the Sequencing and Fragment Analysis Unit of the University of Santiago de Compostela using an ABI 3730×l sequencer (Applied Biosystems, Foster City, CA, USA). Nucleotide sequences were edited and aligned with Chromas Pro v.2.1.4. (Technelysium, South Brisbane, Australia) and BioEdit Sequence Alignment Editor v.7.2.3. (Ibis Biosciences, Carlsbad, CA, USA), respectively. Finally, consensus sequences were searched against the GenBank® database using the Basic Local Alignment Search Tool (BLAST; https://blast.ncbi.nlm.nih.gov/Blast.cgi). Those isolates identified as *C. parvum* by PCR-RFLP of *SSU rRNA* gene were further characterized at the 60-kDa glycoprotein (*GP60*) gene using a previously described nested PCR protocol [35]. After performing sequencing and sequence analysis as previously described, subtypes were categorized using the nomenclature system proposed by Sulaiman et al. [36].

Similarly, those *G. duodenalis* IFAT-positive samples were molecularly characterized at the β-giardin (*bg*), glutamate dehydrogenase (*gdh*) and triosephosphate isomerase (*tpi*) genes as previously described [37–39]. Positive samples were sequenced and analysed as described above. Phylogenetic analysis was conducted using MEGA X software [40].

### 2.4. Statistical analysis

All statistical analyses were performed using the statistical software R [41]. Differences were considered statistically significant at *p* < 0.05. The possible influence of three variables on the presence of each pathogen was assessed by a logistic regression. The independent variables were age ([1] 0–7 days-old, [2] 8–14 days-old, [3] 15–21 days old, [4] >21 days old), faecal consistency ([1] semi-liquid, [2] watery), and seasonality ([1] warm season, [2] cold season). To compare all pairs, the reference categories were changed for the age variable. This analysis was performed with the brglm2 package allowing an estimation and inference from generalized linear models using implicit and explicit bias reduction methods [42].

The analyses for detecting significant variations in the intensity of (oo)cyst elimination were carried out using a multinomial logistic regression; the elimination was classified as 1, 2 and 3 according to the (oo)cyst shedding score of *C. parvum* and *G. duodenalis*, as previously stated. *Eimeria* spp. oocyst excretion was categorized as [1] ≤ 500 opg, [2] > 500–5000 opg, and [3] > 5000 opg [43]. This test was performed with the “multinom” function from the “nnet” package [44].

In addition, the occurrence of pairwise co-infections between the three pathogens was evaluated by testing whether the observed frequency of pairwise co-occurrence was greater or lower than expected given the overall prevalence of a single pathogen at an alpha threshold of 0.05 [45]; this test allows to determine whether a particular pair of pathogens significantly occurred together (positive association), separated (negative association) or at random. The co-occurrence analysis was performed using the “cooccur” package [46]. The relationships between the number of samples positive to each protozoan were represented using an area-proportional Euler diagram, which was fitted using the “eulerr” function from the “eulerr” package [47].

## 3. Results

### 3.1. Prevalence and intensity of (oo)cyst shedding

**Figure 1.**
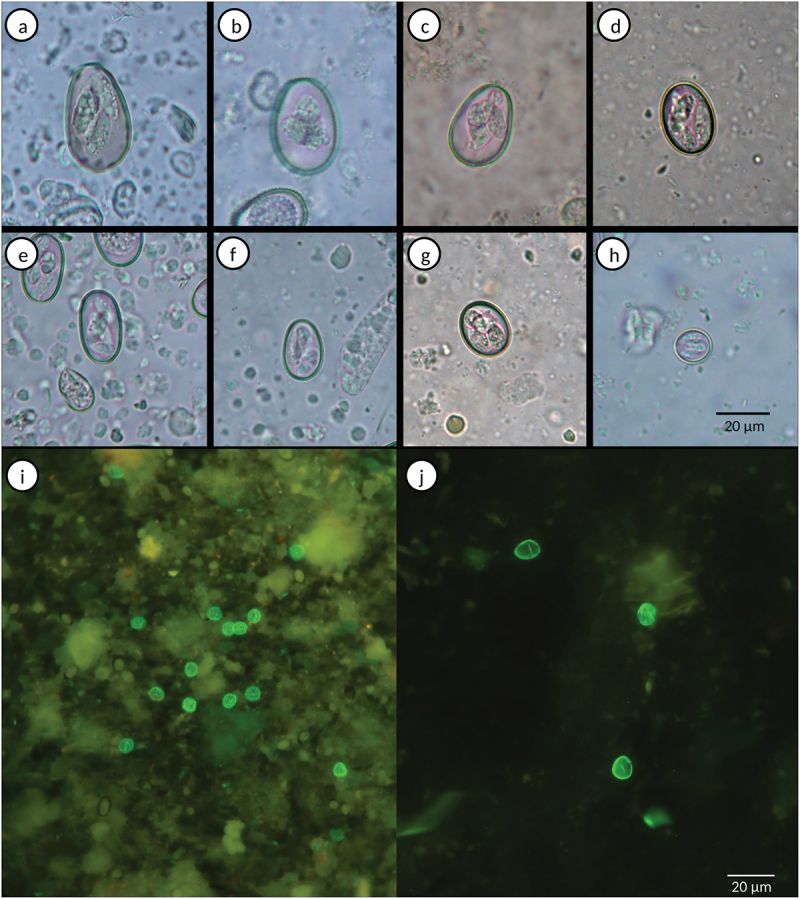
Most prevalent parasitic forms detected in faecal samples from diarrhoeic calves under 1 month old. a) *E. auburnensis* oocyst; b) *E. canadensis* oocyst; c) *E. bovis* oocyst; d) *E. ellipsoidalis* oocyst; e) *E. cylindrica* oocyst; f) *E. alabamensis* oocyst; g) *E. zuernii* oocyst; h) *E. subspherica* oocyst; i) *Cryptosporidium* spp. oocysts; *G. duodenalis* cysts.

At least one of the studied protozoans was detected in 256 faecal samples out of 404 calves analysed (63.4%) (Figure 1). Positive faecal samples were found in 151 out of 221 visited farms (68.4%). The percentage of *Cryptosporidium*-positive samples (53.7%) was higher than that of *G. duodenalis* (12.4%) and *Eimeria* spp. (6.9%). Considering the semi-quantitative evaluation of (oo)cyst shedding, the average *Cryptosporidium* spp. shedding score was higher (2.3; SD 0.8) than the corresponding value obtained for *G. duodenalis* (1.8; SD 0.9). The mean *Eimeria* oocyst output was 25,615.8 opg (SD 83,939.3), ranging from 50 to 434,000 opg; considering the high pathogenic *Eimeria* species, the prevalence of *E. bovis* was higher than that of *E. zuernii*, which showed higher mean oocyst shedding (Table 1).

**Table 1.** Prevalence and (oo)cyst shedding of *C. parvum*, *G. duodenalis* and *Eimeria* spp., including *E. bovis* and *E. zuernii*, according to the age of the calves, faecal consistency and seasonality.

	*Cryptosporidium parvum*		*Giardia duodenalis*		*Eimeria* spp.		*Eimeria bovis*		*Eimeria zuernii*	
	Positives/total (%)	Mean oocyst score (Standard deviation)	Positives/total (%)	Mean cyst score (Standard deviation)	Positives/total (%)	Mean opg (Standard deviation)	Positives/total (%)	Mean opg (Standard deviation)	Positives/total (%)	Mean opg (Standard deviation)
Age										
0–7 days	67/188 (35.6)	2.27 (0.8)	6/188 (3.2)	1.50 (0.8)	0/188 (0)	0 (0)	0/188 (0)	0 (0)	0/188 (0)	0 (0)
8–14 days	97/121 (80.2)	2.47 (0.7)	11/121 (9.2)	1.45 (0.7)	1/121 (0.8)	2,333 (0)	0/121 (0)	0 (0)	0/121 (0)	0 (0)
15–21 days	30/51 (58.8)	2.15 (0.8)	13/51 (25.5)	2.08 (0.9)	4/51 (7.8)	4,862.5 (3,761.3)	3/51 (5.9)	1,219.9 (1,754.8)	3/51 (5.9)	3,142.4 (3,147.4)
>21 days	7/44 (15.9)	1.60 (1.0)	20/44 (45.5)	2.00 (0.9)	23/44 (52.3)	30,237.4 (92,303.1)	21/44 (47.7)	2,602.5 (5,106.1)	17/44 (38.6)	26,481.4 (91,356.1)
Faecal consistency										
Semi-liquid	63/155 (40.6)	2.30 (0.8)	28/155 (18.1)	1.89 (0.9)	14/155 (9.0)	11,760.1 (29,993.2)	11/155 (7.1)	689.5 (1,254.1)	9/155 (5.8)	3,157.6 (4,878.8)
Watery	138/249 (55.4)	2.41 (0.7)	22/149 (14.8)	1.77 (0.9)	14/249 (5.6)	39,471.4 (99,243.1)	13/249 (5.2)	3,902.2 (6,160.3)	11/249 (4.4)	39,199.3 (113,279.8)
Seasonality										
Warm season	129/260 (49.6)	2.30 (0.8)	36/260 (13.8)	1.72 (0.8)	16/260 (6.2)	37,350.6 (109,500.1)	14/260 (5.4)	2,485.8 (5,479.6)	12/260 (4.6)	33,141.5 (108,551.8)
Cold season	72/144 (50.0)	2.51 (0.7)	14/144 (9.7)	2.14 (0.9)	12/144 (8.3)	9,969 (21,826.9)	10/144 (6.9)	2,351.2 (3,974.5)	8/144 (5.6)	7,739.1 (17,840.1)
Total	201/404 (49.8)	2.37 (0.8)	50/404 (12.4)	1.84 (0.9)	28/404 (6.9)	25,615.8 (83,939.3)	24/404 (5.9)	2,429.7 (4,812.2)	20/404 (5.0)	22,989.5 (84,275.1)

opg: oocysts per gram of faeces.

### 3.2. Species identification and genotyping and subtyping analyses

The amplification of a fragment of the *SSU rRNA* gene of *Cryptosporidium* spp. was obtained in 205 out the 217 IFAT-positive samples (94.5%). The RFLP pattern enabled the identification of three *Cryptosporidium* species: *C. parvum* was the predominant species (201/205; 98.0%), being detected in animals from 118 farms (53.4%). In contrast, *C. bovis* and *C. ryanae* were only found in two samples each (2/205; 1.0%) from four different farms (1.8%). The sequence analysis allowed us to confirm the RFLP results. Thus, the sequences of those samples identified as *C. bovis* and *C. ryanae* were identical to the sequences deposited in the GenBank^Ⓡ^ database under the accession numbers MH028031 and KY711520, respectively. A single *Cryptosporidium* species was identified in all farms where two or more animals were analysed, except for one herd where both *C. parvum* and *C. bovis* were concurrently identified.

Subtyping at the *GP60* gene was successful in 195 out of 201 samples (97.0%) in which *C. parvum* was identified by PCR-RFLP. The identified *C. parvum* subtypes, the number of positive samples and their percentage of similarity when compared to the GenBank^Ⓡ^ reference sequences are summarized in Table 2. A total of 11 *C. parvum GP60* subtypes belonging to the IIa allelic family were identified after sequence analysis, being the subtype IIaA15G2R1 the most common one (155/195; 77.1%), followed by the IIaA16G3R1 (24/195; 11.9%); the remaining subtypes were only occasionally found (Table 2). A single *C. parvum* subtype was identified in all farms except in two in which the subtypes IIaA15G2R1-IIaA17G3R1 and IIaA15G2R1-IIaA16G3R1 were found.

**Table 2.** *C. parvum* 60-kDa glycoprotein (GP60) subtypes identified in faecal samples from diarrhoeic calves younger than one month from north-western Spain.

*GP60* subtype	n (% of *C. parvum*-positive samples)	GenBank® reference sequence (n)	Identity (%)
IIaA15G2R1	155 (77.1%)	MT010357 (151) MT010357 (3) MT010357 (1)	100% 99.9% 99.7%
IIaA16G3R1	24 (11.9%)	JQ362492 (24)	100%
IIaA16G1R1	3 (1.5%)	MT010358 (3)	100%
IIaA17G3R1	3 (1.5%)	GU214359 (3)	100%
IIaA13G2R1	2 (1.0%)	MN815775 (2)	100%
IIaA17R1	2 (1.0%)	LT556066 (2)	99.9%
IIaA18G3R1	2 (1.0%)	MG516786 (2)	100%
IIaA14G1R1	1 (0.5%)	MK034687 (1)	100%
IIaA14G2R1	1 (0.5%)	KF128738 (1)	100%
IIaA17G2R1	1 (0.5%)	MT010359 (1)	100%
IIaA17G4R1	1 (0.5%)	MF142039 (1)	100%

In those six faecal samples for which the *GP60* amplification was not achieved, molecular identification by sequencing of the *SSU rRNA* gene was performed allowing the identification of *C. parvum* in all of them. Five isolates were identical to the *C. parvum* sequence deposited in the GenBank^Ⓡ^ database under the accession number MT071829, whereas the remaining isolate exhibited a single nucleotide transition when compared to the sequence MT648442.

PCR products of at least one of the three *Giardia* genes analysed (*gdh*, *bg* and *tpi*) were obtained in seven out of 50 IFAT-positive samples (14.0%); *G. duodenalis* assemblage E was identified in all of them (Supplementary Figures S1–3). Thus, partial sequences of the *gdh* gene were obtained from five samples. Two sequences showed a similarity of 100% with the sequence deposited in the GenBank^Ⓡ^ database under the accession number MK561347 and the remaining three sequences exhibited one nucleotide variation (99.8% identity) with the same reference sequence. At the *bg* gene, three partial sequences were obtained, being two of them identical to the deposited sequence LC484286 and the remaining one exhibited two nucleotide variations (99.7% identity) regarding the same reference sequence. Finally, partial sequences of the *tpi* gene were obtained from three samples, which exhibited one or two nucleotide differences (99.6–99.8% identity) with the sequence AB569406.

All the novel partial sequences identified in this study were deposited in the GenBank^Ⓡ^ database under accession numbers OR157865, OR177062-OR177069 and OR238467-OR238477.

**Figure 2.**
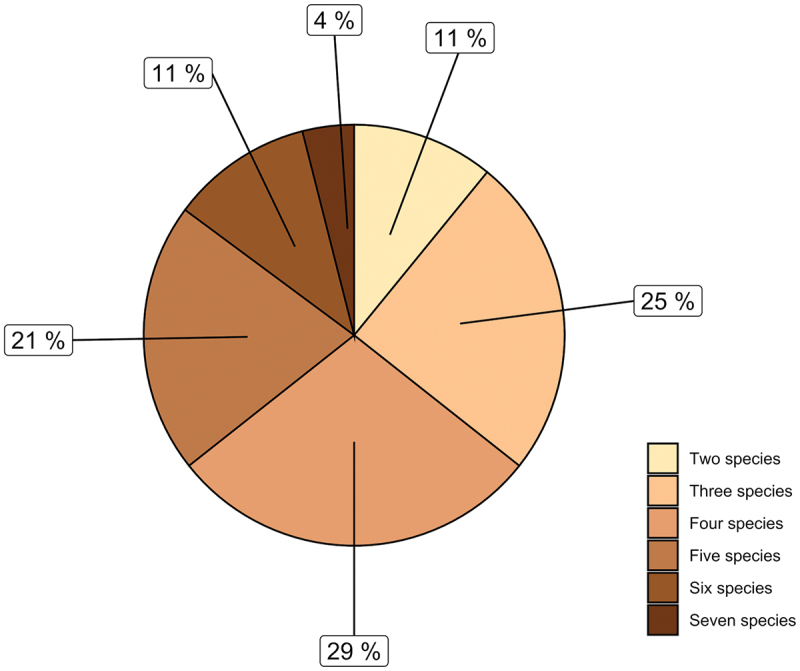
Mixed infections with different *Eimeria* species in diarrhoeic calves under 1 month old.

The species of *Eimeria* were identified in all the microscopically positive samples. The morphometrical examination of oocysts allowed the identification of 10 *Eimeria* species (Figure 1), with *E. ellipsoidalis*, *E. bovis* and *E. zuernii* being the most prevalent (Table 3). Mixed infections with different *Eimeria* species (up to seven) were detected in all the positive samples (Table 3; Figure 2).

**Table 3.** Prevalence of *Eimeria* species identified in faecal samples from diarrhoeic calves younger than one month from north-western Spain.

*Eimeria* species	Prevalence (%)	Positives/*Eimeria*-positive(%)	Co-infections (n)									
			*E.e*	*E.b*	*E.z*	*E.a*	*E.c*	*E.s*	*E.ca*	*E.al*	*E.w*	*E.p*
*E. ellipsoidalis (E.e)*	27/404 (6.7)	27/28 (96.4)		23	19	12	9	7	6	6	2	1
*E. bovis (E.b)*	24/404 (5.9)	24/28 (85.7)			17	11	7	6	6	5	2	1
*E. zuernii (E.z)*	20/404 (5.0)	20/28 (71.4)				8	8	7	4	4	1	1
*E. auburnensis (E.a)*	12/404 (3.0)	12/28 (42.9)					3	4	3	3	2	1
*E. cylindrica (E.c)*	9/404 (2.2)	9/28 (32.1)						–	–	3	–	–
*E. subspherica (E.s)*	7/404 (1.7)	7/28 (25.0)							–	1	–	–
*E. canadensis (E.ca)*	6/404 (1.5)	6/28 (21.4)								1	1	1
*E. alabamensis (E.al)*	6/404 (1.5)	6/28 (21.4)									–	–
*E. wyomingensis (E.w)*	2/404 (0.5)	2/28 (7.1)										1
*E. pellita (E.p)*	1/404 (0.2)	1/28 (3.6)										

### 3.3. Associations between protozoan parasites

**Figure 3.**
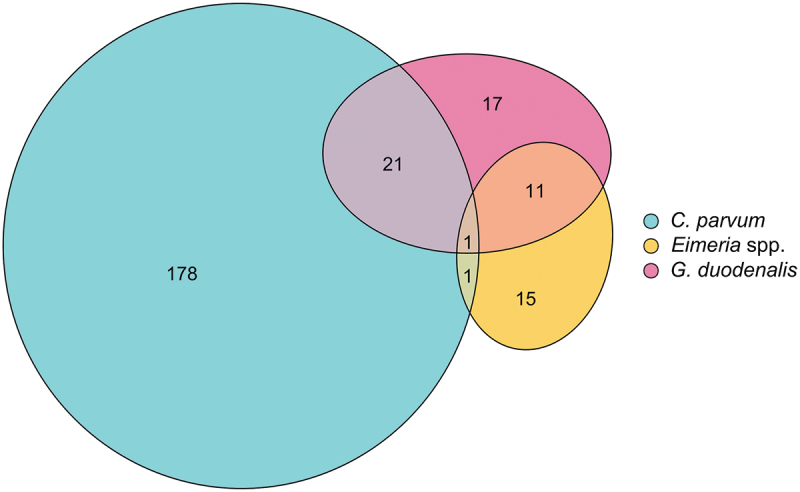
Euler’s diagram representing the number of mono-specific and mixed infections between *C. parvum*, *G. duodenalis* and *Eimeria* spp. in diarrhoeic calves.

**Figure 4.**
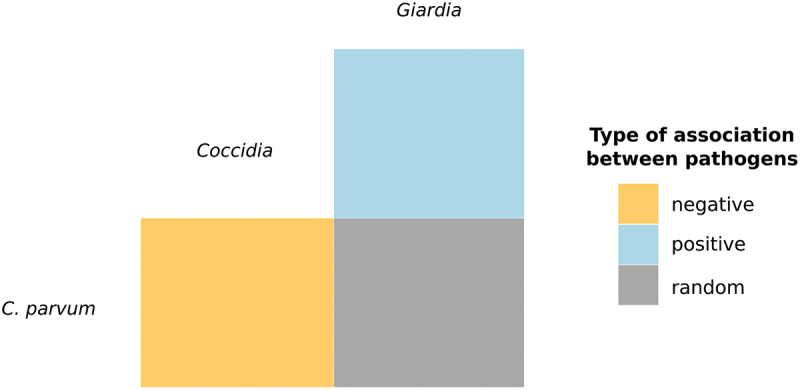
Heat map showing positive, negative and random species associations determined by the probabilistic co-occurrence model for *C. parvum*, *G. duodenalis* and *Eimeria* spp. in diarrhoeic calves from north-western Spain.

When assessing the associations between the three genera of protozoans, only those samples molecularly identified as *C. parvum* were included. Samples in which *C. bovis* and *C. ryanae* were identified were not analysed because of their low prevalence and limited clinical relevance. In addition, no particular *Eimeria* species were included in the analyses since moderate to high pathogenic species were detected in all samples. Regarding the 244 positive samples, a single protozoan genus was detected in 210 (86.1%) samples (Figure 3). Mixed infections were found in a high percentage of *G. duodenalis* (33/50; 66.0%) and *Eimeria* spp. (13/28; 46.4%) positive samples, whereas co-infections with *C. parvum* were detected in a lower percentage (23/201; 11.4%). Infections integrated by two different parasites were found in 33 samples (13.5%), being *C. parvum*/*G. duodenalis* (21/33; 63.6%) and *G. duodenalis*/*Eimeria* spp. (11/33; 33.3%) the most prevalent associations. The co-infection of *C. parvum/Eimeria* spp. was only observed in one sample (1/33; 3.0%). Triple infections were only detected in a single sample (1/244; 0.4%) (Figure 3). Moreover, the probabilistic analysis of co-occurrences revealed a significant positive association between *G. duodenalis* and *Eimeria* spp. (*p* < 0.001), whereas the presence of *C. parvum* was significantly related to the absence of *Eimeria* spp. (*p* < 0.001) (Figure 4).

### 3.4. Influence of factors on the prevalence and (oo)cyst shedding of protozoan infections

**Figure 5.**
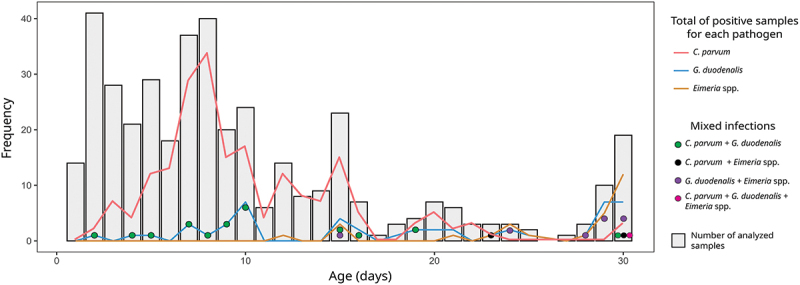
Number of samples positive to *C. parvum*, *G. duodenalis* and *Eimeria* spp. and their co-infections when considering the age of the calves.

Most diarrhoeic samples (306/404; 75.7%) were collected in calves under 15 days of age and the average age of the sampled calves was 10.17 ± 7.87 days (Figure 5). When analysing the possible influence of age on the percentage of positives, *C. parvum* was found to be the most prevalent parasite during the first 21 days of life, being its prevalence significantly highest in calves between 8 and 14 days of age (Table 1; Figure 5; Supplementary Table S1). The multinomial logistic regression identified that *C. parvum* oocyst shedding was significantly lower in animals aged 15–21 days (OR = 0.37 [CI95% 0.18–0.77]; *p*  = 0.031, comparing oocyst shedding scores [2] and [3]) and older than 21 days (OR = 0.16 [CI95% 0.03–0.81]; *p*  = 0.027, comparing oocyst shedding scores 1 and 3) than in calves aged 8–14 days. *C. bovis* and *C. ryanae* were only found in calves aged 10–18 days. In contrast, *G. duodenalis* and *Eimeria* infections were mainly established after 21 days. In all age groups, *G. duodenalis* was detected and its prevalence significantly increased with age (Table 1; Figure 5; Supplementary Table S2). No calves younger than one week old were positive to *Eimeria* spp., and the highly pathogenic species *E. bovis* and *E. zuernii* were not found in animals under 14 days. As observed for *G. duodenalis*, the percentage of calves infected with coccidia significantly increased with age; in fact, animals aged four weeks had a 15.6 to 10^8^-fold increased risk of being positive to *Eimeria* spp. than younger ones (Table 1; Figure 5; Supplementary Table S3). No significant differences in the intensity of (oo)cyst excretion was found for *G. duodenalis* and *Eimeria* spp.

Regarding the possible influence of the faecal consistency on the prevalence and (oo)cyst shedding of each protozoan, the highest percentage of *C. parvum* positives and *Eimeria* spp. opg counts was observed in watery faeces (Table 1). However, significant differences were only found for the prevalence; thus, the probability of finding *C. parvum* oocysts was 1.6-fold higher in watery faeces (Supplementary Table S1). In contrast, the percentage of positives to *G. duodenalis* and *Eimeria* spp. (including *E. bovis* and *E. zuernii*) was higher in semi-liquid than in watery samples (Table 1), although differences were not significant (Supplementary Tables S2 and 3). The intensity of *G. duodenalis* cyst excretion was highest in semi-liquid samples (*p*  > 0.05) whereas *Eimeria* oocyst output was highest in watery samples (*p*  > 0.05) (Table 1). Finally, no significant differences in the prevalence and (oo)cyst elimination were detected when considering the seasonality (Table 1, Supplementary Tables S1–3).

Regarding co-occurrences, the percentage of samples showing mixed infections increased with age, from 3.2% in the youngest animals to 31.8% in the oldest. The association *C. parvum/G. duodenalis* was more common in calves younger than 21 days and *G. duodenalis/Eimeria* spp. in those older than 21 days (Figure 5). The co-infections *C. parvum/Eimeria* spp. and *C. parvum/G. duodenalis/Eimeria* spp. were only detected in two animals aged 23 and 30 days old, respectively (Figure 5).

## 4. Discussion

### 4.1. Prevalence of the three protozoa genera

Our results confirm that *C. parvum* is the major protozoan involved in calves with diarrhoea under one month of age from north-western Spain, showing high individual prevalences. Previous investigations demonstrated that *Cryptosporidium* is a very frequent parasite in ruminant farms from the study area, including both healthy and diarrhoeic animals [4,7,11,18,20,29]; in fact, a longitudinal study performed in the same region showed that all the calves got infected with *C. parvum* in their first 30 days of life [19]. It is worth noting that our findings are similar to those previously reported in pre-weaned calves with diarrhoea from Spain in the last 25 years [7,8,17,22], even though drugs for preventing cryptosporidiosis were available during that period, revealing that the control of this protozoan in cattle farms is still a challenge for farmers and veterinarians in our country. In this regard, *C. parvum* is considered, together with rotavirus, the most prevalent enteropathogen in diarrhoeic calves [14,17,21]. Moreover, *C. bovis* and *C. ryanae* were also identified, although in a limited number of diarrhoeic samples (*n* = 4). Since these host-specific species are considered non-pathogenic [9], the observed clinical signs may be the result of mixed infections with other enteropathogens. In fact, these species were found in co-infection with other protozoans in two samples: one sample positive to *C. bovis* was also positive to *G. duodenalis* whereas *G. duodenalis* and *Eimeria* spp. (*E. bovis*, *E. canadensis*, *E. ellipsoidalis* and *E. zuernii*) were also found in one sample positive to *C. ryanae*.

There is wide evidence that *G. duodenalis* is common in subclinical or asymptomatic cattle [12,22], even in the study area [11,20]. However, most investigations on the aetiology of NCD did not assess the role of *G. duodenalis* infections and thus its prevalence and pathogenicity are not clear [48]. Our work provides information on the presence of *G. duodenalis* in diarrhoeic calves aged less than 30 days, which is especially limited. Thus, our results reveal that *G. duodenalis* is frequent in NCD samples (12.4%), which is consistent with previous reports in suckling diarrhoeic calves from Spain [22] and Austria [49], with values ranging from 9.8% to 10.6%, respectively. In contrast, a lower percentage of infection (3.5%) was found in diarrhoeic calves under one month of age from Germany [14], whereas higher prevalences were detected in Asian and American countries such as Iran and Brazil (21.3% and 25.6%, respectively) [24,50]. Although Castro-Hermida et al. [11] only found a significant association between parasitization by *G. duodenalis* and the presence of diarrhoeic faeces in 1–5 month-old calves, more robust data is needed to completely elucidate the role of *G. duodenalis* on the onset of diarrhoea outbreaks. Several case and experimental studies [48,51] concluded that *G. duodenalis* contributes to NCD and a recent meta-analysis showed that diarrhoeic calves have a 2.6-fold higher probability of being infected with *G. duodenalis* than healthy animals [12]. All these findings together with our results suggest that this protozoan should be routinely included in the diagnosis of NCD outbreaks affecting calves >15 days old.

Since infection with *Eimeria* spp. is especially prevalent in calves from 3 weeks up to 6 months of age [3], global information on its presence in diarrhoeic calves under a month of age is quite scarce, especially data including species identification at the individual level. In our study, the percentage of calves under one month old positive to *Eimeria* spp. was low (6.9%) and similar to that previously found in diarrhoeic calves of the same age (4.5%) [14]. In the present study, ten *Eimeria* species were identified, always in mixed infections of up to seven different species. These results are in line with previous studies which indicate that infections with a single *Eimeria* species are uncommon, even in asymptomatic and in both pre-weaned and post-weaned animals [52,53]. Highly (*E. zuernii* and *E. bovis*) and mildly (*E. ellipsoidalis*) pathogenic *Eimeria* species [16] were the most prevalent and abundant, which may be related to the fact that all the sampled animals were diarrhoeic.

### 4.2. Pathogen associations and influence of age, faecal consistency and season on the prevalence

The current study provides new data on the presence and associations of the three most common genera of protozoans in diarrhoeic calves younger than one month, where NCD is especially common [25,49]. Our data revealed that mixed infections among these three parasites were not very common. Particularly, concurrent infections between *C. parvum* and *Eimeria* spp. were very scarce (0.25%) and significantly and negatively associated, which is in line with previous results (0.26%) reported in diarrhoeic calves from Germany [14]. This may be because infections with these parasites are age-related. Thus, a significant peak in the prevalence of *C. parvum* was observed in diarrhoeic calves between 8 and 14 days of age, followed by a significant decrease in the percentage of positive samples in older animals. This pattern is consistent with previous studies [14,21,54] and correlates with the intensity of oocyst excretion, which decreased significantly after the second week of life. This age-related oocyst shedding pattern is in line with previous longitudinal studies reporting an increase in the oocyst excretion, peaking at around 12 days of age and decreasing after that age [55,56]. In contrast, the prevalence and the intensity of (oo)cyst excretion of *Eimeria* spp. and *G. duodenalis* showed an increasing tendency with age, although it was only significant for the prevalence. Thus, *Eimeria* oocysts are mainly detected in calves older than 21 days since the prepatent period of a number of species, including the most pathogenic ones, is longer than 15 days [16]; in this regard, Gillhuber et al. [14] also observed a progressive increase from 2.8% in the first three weeks of life to 29% in the fourth one, although the highest prevalence (52.6%) was found in animals aged 61–90 days. Although the prepatent period for *G. duodenalis* is shorter, since cysts can be detected in animals under 7 days old, its prevalence also increases significantly with age [14,49,50]. In fact, Gillhuber et al. [14] observed that the odds of being infected with *G. duodenalis* increased up to 8 times from about 12 days to 30 days, peaking at 61–90 days of life. These results can explain why the co-occurrence analysis also showed a significantly positive association between *G. duodenalis* and *Eimeria* spp.; in fact, 42.9% and 24.0% of all the positive samples to *Eimeria* spp. and *G. duodenalis*, respectively, were mixed infections between these two protozoans (see Figure 3). In addition, our results might reflect a synergistic interaction between these two protozoans, so that the presence of one of them facilitates the infection with the other one. It has also been suggested that infections with *Cryptosporidium* spp. may act as a predisposing factor for subsequent infections with *G. duodenalis* or *Eimeria* spp [3,14,57]. Nevertheless, further longitudinal studies are needed to assess these hypotheses.

Regarding the possible relationship between the infection with each of these agents and faecal consistency, only the presence of *C. parvum* oocysts was significantly positively related to watery faecal samples. This result could be due to the high pathogenicity of *C. parvum*, which has been associated with the excretion of watery faeces [57–59]. Although a significant relationship between average *C. parvum* shedding score and faecal consistency was not found in this study, other investigations detected that the onset of diarrhoea was significantly related to high levels of oocyst shedding [59–61]. In contrast, although the prevalence and intensity of cyst shedding of *G. duodenalis* was higher in semi-liquid than in watery samples, no significant differences were found. These results are consistent with other investigations reporting that the consistency of faeces was also not associated with an increased risk of *G. duodenalis* infection [22,62,63]. In this regard, it is difficult to find a correlation between faecal consistency and infection and cyst output through a one sampling study design because it was reported that this protozoan causes chronic intermittent diarrhoea in calves [62]. However, other studies have described a relation between faecal consistency and prevalence and cyst excretion [13,57]. Although watery faeces are often associated with *Eimeria* infections in calves [64], an association between the prevalence and intensity of excretion of *Eimeria* oocysts and faecal consistency was not detected in our study; in this regard, other investigations have found that the severity of diarrhoea is not related to oocyst shedding [65–67]. In contrast, significant higher oocyst counts have been also found in watery faeces [68–70]. Considering all these discrepant results, more studies on this issue are needed to elucidate whether faecal consistency is related to prevalence and (oo)cyst shedding of these three protozoans. It is also worth noting that non-diarrhoeic calves were not included in our study, and this could bias the results of the statistical analysis.

Finally, in this study, neither the prevalence nor the intensity of excretion of any of the studied parasites showed any seasonality. Higher prevalence and/or (oo)cyst output values of *C. parvum* [71,72], *G. duodenalis* [43,71,73,74] and *Eimeria* spp. [43] have been described during certain seasons, mainly related to high rainfall, warm temperatures or the number of births [71]. Our results may be due to the absence of significant climatic variations throughout the year as well as the lack of a particular calving season in the study area and only diarrhoeic calves were sampled [18].

### 4.3. Genotyping and subtyping analyses

The subtype analysis of the *C. parvum* isolates revealed the presence of 11 subtypes which belonged to the allelic family IIa; this is the major allelic family found in calves from Spain [4,7,8], as well as in cattle worldwide, although other families such as IIb, IId and IIj have also been identified in cattle [4,5]. In addition, our data showed a higher *C. parvum* genetic diversity than that previously observed in different northern Spanish regions by Quílez et al. [8] and Díaz et al. [7], where up to seven different subtypes were found. Nevertheless, these differences may be related to the smaller number of positive samples (*n* = 30–149) analysed in those studies. In line with previous investigations in Europe, the zoonotic and hypertransmissible IIaA15G2R1 subtype was the predominant one in the studied animals [7,8,49,54,75–77]. This subtype is currently also considered the most common *C. parvum* subtype in humans, playing an important role in human cases of cryptosporidiosis globally [5,78]. Furthermore, this study provides the first report of the subtypes IIaA13G2R1, IIaA14G1R1, IIaA14G2R1, IIaA16G1R1, IIaA17G3R1, IIaA17G4R1 and IIaA17R1 in cattle from Spain; all of them have also been detected in humans [79,80]. These results, together with the predominance of the subtype IIaA15G2R1, suggest the existence of a zoonotic risk and the importance of subtyping tools for tracking both animal and human cryptosporidiosis outbreaks. Finally, it must be pointed out that only animals belonging to two farms were infected by two *C. parvum* subtypes, which is consistent with previous studies [54,81].

The molecular analysis of *G. duodenalis* isolates revealed the existence of only the assemblage E, which is the most common one in cattle [12]. It is worth noting that only a very small number of IFAT-positive samples could be identified to the assemblage level, which may be related to the huge genetic diversity detected within assemblage E. In fact, a recent investigation identified more than 25 unique assemblage E sequences in pre-weaned calves from the USA [82] and similar results have been reported in other countries [10,83,84]. This high sequence polymorphism may denote mutations in the primer binding region leading to sequence amplification failure. Although assemblage E is not considered important from a zoonotic point of view, it has been sporadically detected in humans [85] and, thus, our results may have public health implications. The absence of the zoonotic assemblages A or B may be due to the low efficiency of molecular genotyping or to the presence of mixed infections with assemblage E. In fact, both subtypes have been detected in small numbers in previous studies performed in cattle from the same region and from northern Portugal, close to the study area [20,86]. In this regard, a recent investigation revealed a low sensitivity of Sanger sequencing in detecting assemblages present in low abundances, since next generation sequencing techniques allowed the identification of six-times more mixed infections [82]. In this study, the sole identification of *G. duodenalis* assemblage E in diarrhoeic calves might indicate an assemblage-specific pathogenicity, as previously suggested by Gillhuber et al. [14]. These authors reported that *G. duodenalis* assemblage E might contribute to diarrhoea in calves, as it was the only pathogen found in a number of diarrhoeic faecal samples, whereas assemblage A was only found in co-infections with *Cryptosporidium* spp. or *Eimeria* spp. Therefore, more robust data is needed for a better understanding of *G. duodenalis* assemblage dynamics in cattle, especially in diarrhoeic calves.

## Conclusions

Our study revealed that *C. parvum* is clearly the most prevalent and widely distributed protozoan pathogen among diarrhoeic calves younger than one month; *Eimeria* spp. and *G. duodenalis* are present in a lower but non-negligible number of cases. Concurrent infections among these parasites were not common because these parasites show a strong age-dependence; however, a significant positive association was detected between *G. duodenalis* and *Eimeria* spp., especially in the fourth week of life. Our results also pose a public health risk since a significant percentage of animals were infected with *C. parvum* subtypes previously detected in humans, although their contribution to human infection (especially among vulnerable populations) must be further explored. Further studies are needed for determining the role of these parasites in co-infections with other bacterial and viral enteropathogens and their implications in the pathogenicity of NCD.

## Supplementary Material

Sup Fig 1.tiff

Sup Fig 2.tiff

Sup Fig 3.tiff

Supplementary Tables_R1.docx
