# Supplementary figures and images for: Prevalence of Cryptosporidium parvum, Giardia duodenalis and Eimeria spp. in diarrhoeic suckling calves from north-western Spain and analysis of their interactions
Source: Int J Vet Sci Med. 2025 Jan 9;13(1):1–14. doi: 10.1080/23144599.2024.2447172 (PMC11721762; doi:10.1080/23144599.2024.2447172)

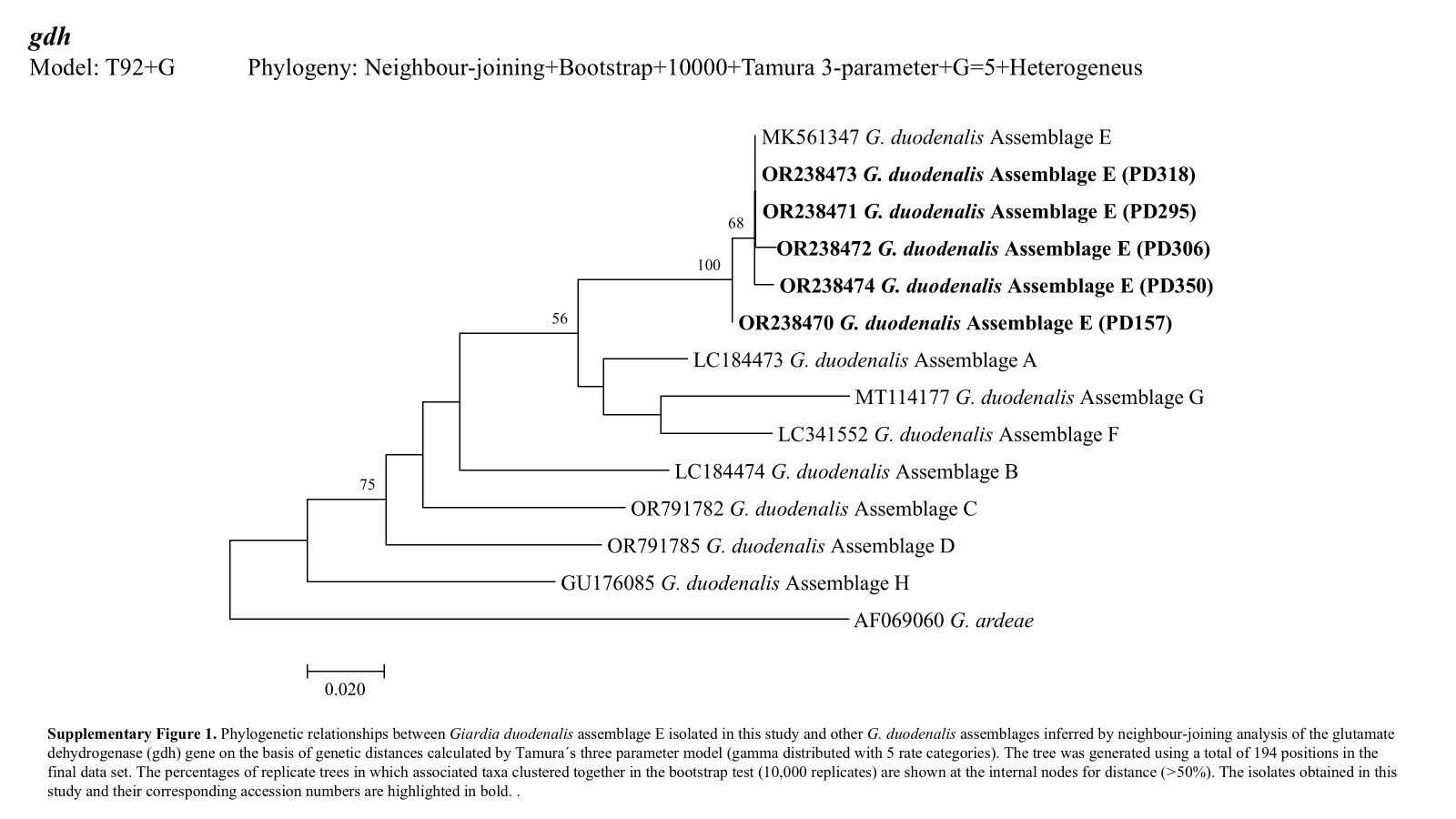

Supplement: Sup Fig 1.tiff [file TVSM_A_2447172_SM7145.tiff]

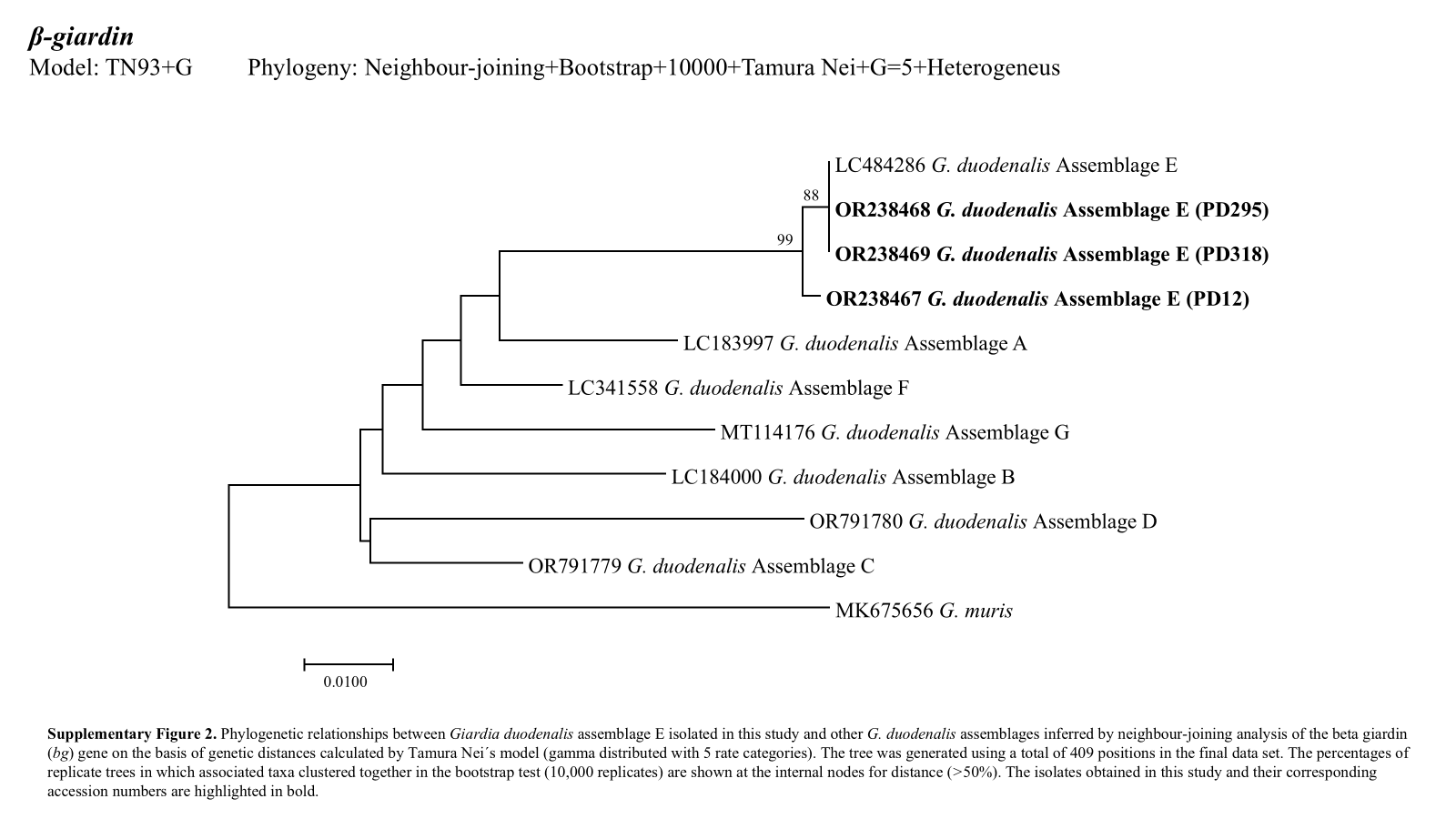

Supplement: Sup Fig 2.tiff [file TVSM_A_2447172_SM7144.tiff]

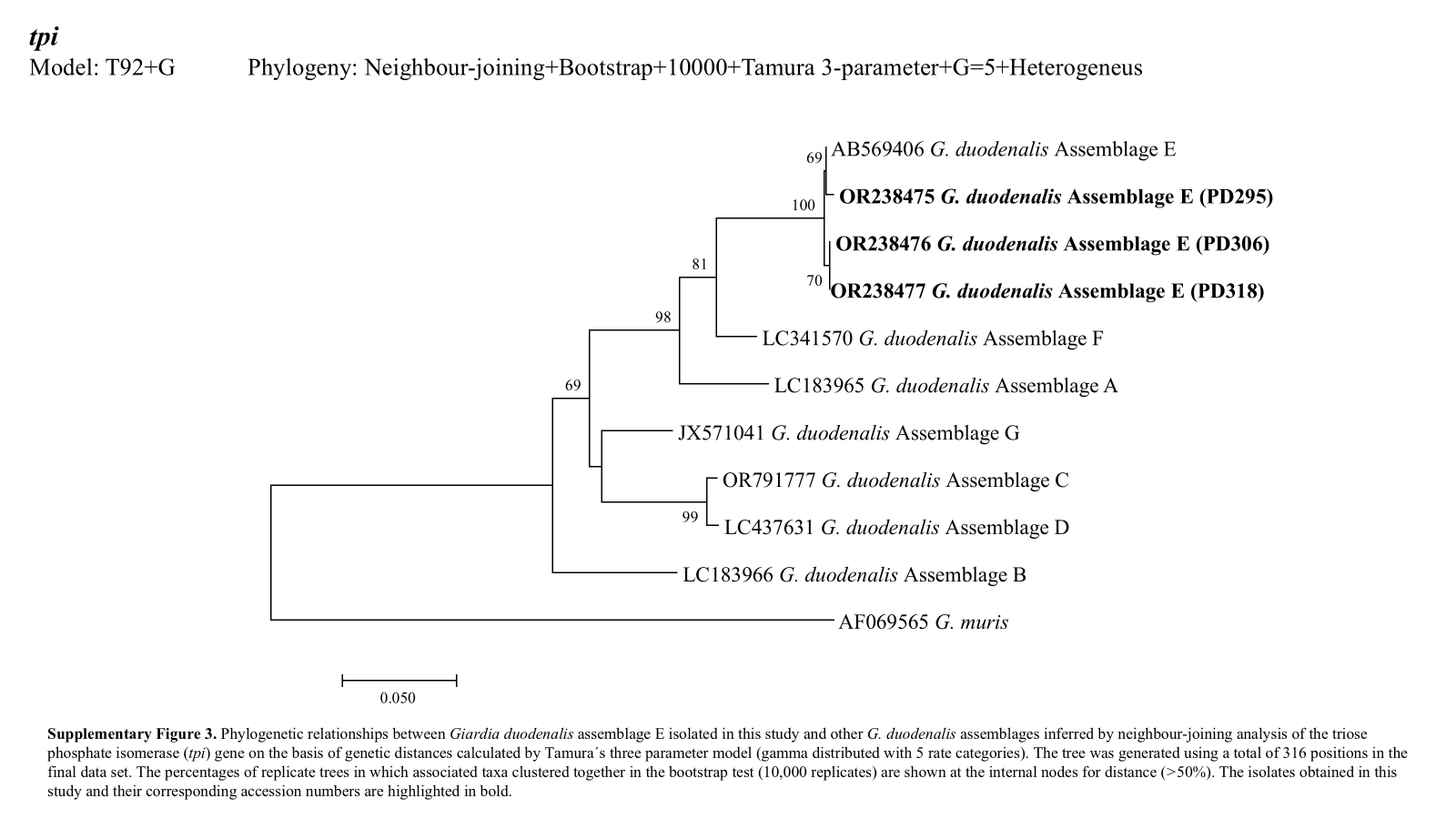

Supplement: Sup Fig 3.tiff [file TVSM_A_2447172_SM7143.tiff]
