# Supplementary material for: Prevalence of Cryptosporidium parvum, Giardia duodenalis and Eimeria spp. in diarrhoeic suckling calves from north-western Spain and analysis of their interactions
Source: Int J Vet Sci Med. 2025 Jan 9;13(1):1–14. doi: 10.1080/23144599.2024.2447172 (PMC11721762; doi:10.1080/23144599.2024.2447172)
Supplement: Supplementary Tables_R1.docx [file TVSM_A_2447172_SM7142.docx]

Supplementary Table 1. Logistic regression model for the prevalence of *Cryptosporidium* *parvum* in diarrhoeic calves younger than 1 month-old from Spain

|  | **Estimate** | **Z value** | ***p* value** | **OR**  **(CI 95%)** | **Estimate** | **Z value** | ***p* value** | **OR**  **(CI 95%)** | **Estimate** | **Z value** | ***p* value** | **OR**  **(CI 95%)** |
| --- | --- | --- | --- | --- | --- | --- | --- | --- | --- | --- | --- | --- |
| **Age** | | | | | | | | | | | | |
| **0-7 days old** | ***** | ***** | ***** | ***** |  |  |  |  |  |  |  |  |
| **8-14 days old** | **2.035** | **7.294** | **< 0.001** | **7.65**  **(4.49-13.45)** | ***** | ***** | ***** | ***** |  |  |  |  |
| **15-21 days old** | **1.037** | **3.129** | **0.002** | **2.82**  **(1.48-5.46)** | **-0.998** | **-2.677** | **0.007** | **0.37**  **(0.18-0.77)** | ***** | ***** | ***** | ***** |
| **> 21 days old** | **-1.006** | **-2.274** | **0.023** | **0.37**  **(0.14-0.82)** | **-3.041** | **-6.393** | **<0.001** | **0.05**  **(0.02-0.12)** | **-2.043** | **-4.030** | **<0.001** | **0.13**  **(0.05-0.34)** |
| **Season** | | | | | | | | | | | | |
| **Cold** | ***** | ***** | ***** | ***** |  |  |  |  |  |  |  |  |
| **Warm** | **-0.189** | **-0.784** | **0.432** | **0.83**  **(0.51-1.32)** |  |  |  |  |  |  |  |  |
| **Faecal consistency** | | | | | | | | | | | | |
| **Semi-liquid** | ***** | ***** | ***** | ***** |  |  |  |  |  |  |  |  |
| **Watery** | **0.496** | **2.093** | **0.036** | **1.64**  **(1.03-2.61)** |  |  |  |  |  |  |  |  |

* Reference Category

|  | **Estimate** | **Z value** | ***p* value** | **OR**  **(CI 95%)** | **Estimate** | **Z value** | ***p* value** | **OR**  **(CI 95%)** | **Estimate** | **Z value** | ***p* value** | **OR**  **(CI 95%)** |
| --- | --- | --- | --- | --- | --- | --- | --- | --- | --- | --- | --- | --- |
| **Age** | | | | | | | | | | | | |
| **0-7 days old** | ***** | ***** | ***** | ***** |  |  |  |  |  |  |  |  |
| **8-14 days old** | **1.177** | **2.242** | **0.025** | **3.25**  **(1.19-9.71)** | ***** | ***** | ***** | ***** |  |  |  |  |
| **15-21 days old** | **2.227** | **0.537** | **<0.001** | **11.56**  **(4.18-35.51)** | **1.270** | **2.729** | **0.006** | **3.56**  **(1.43-9.03)** | ***** | ***** | ***** | ***** |
| **> 21 days old** | **3.201** | **0.529** | **<0.001** | **24.56**  **(9.35-73.64)** | **2.024** | **4.531** | **<0.001** | **7.57**  **(3.21-18.70)** | **0.754** | **1.664** | **0.096** | **2.13**  **(0.88-5.25)** |
| **Season** | | | | | | | | | | | | |
| **Cold** | ***** | ***** | ***** | ***** |  |  |  |  |  |  |  |  |
| **Warm** | **-0.563** | **-1.505** | **0.132** | **0.57**  **(0.27-1.16)** |  |  |  |  |  |  |  |  |
| **Faecal consistency** | | | | | | | | | | | | |
| **Semi-liquid** | ***** | ***** | ***** | ***** |  |  |  |  |  |  |  |  |
| **Watery** | **-0.571** | **-1.682** | **0.093** | **0.56**  **(0.29-1.10)** |  |  |  |  |  |  |  |  |

Supplementary Table 2. Logistic regression model for the prevalence of *Giardia duodenalis* in diarrhoeic calves younger than 1 month-old from Spain.

* Reference Category

Supplementary Table 3. Logistic regression model for the prevalence of *Eimeria* spp. in diarrhoeic calves younger than 1 month-old from Spain

|  | **Estimate** | **Z value** | ***p* value** | **OR**  **CI 95%)** | **Estimate** | **Z value** | ***p* value** | **OR**  **(CI 95%)** | **Estimate** | **Z value** | ***p* value** | **OR**  **(CI 95%)** |
| --- | --- | --- | --- | --- | --- | --- | --- | --- | --- | --- | --- | --- |
| **Age** | | | | | | | | | | | | |
| **0-7 days old** | ***** | ***** | ***** | ***** |  |  |  |  |  |  |  |  |
| **8-14 days old** | **15.745** | **0.012** | **0.990** | **6.8^6^**  **(5.8^-23^-8.1^143^** | ***** | ***** | ***** | ***** |  |  |  |  |
| **15-21 days old** | **17.976** | **0.014** | **0.989** | **6.4^7^**  **(7.1^-23^ – 3.2^84^)** | **2.231** | **1.959** | **0.051** | **9.31**  **(1.31-186.18)** | ***** | ***** | ***** | ***** |
| **> 21 days old** | **20.723** | **0.016** | **0.987** | **10.0^8^**  **(8.4^-21^-1.2^146^)** | **4.979** | **1.065** | **<0.001** | **145.29**  **(27.21-2715.85)** | **2.748** | **4.302** | **<0.001** | **15.60**  **(4.93-63.02)** |
| **Season** | | | | | | | | | | | | |
| **Cold** | ***** | ***** | ***** | ***** |  |  |  |  |  |  |  |  |
| **Warm** | **0.630** | **1.146** | **0.252** | **1.87**  **(0.65-5.75)** |  |  |  |  |  |  |  |  |
| **Faecal consistency** | | | | | | | | | | | | |
| **Semi-liquid** | ***** | ***** | ***** | ***** |  |  |  |  |  |  |  |  |
| **Watery** | **0.118** | **0.226** | **0.821** | **1.12**  **(0.41-3.17)** |  |  |  |  |  |  |  |  |

* Reference Category
